# New Robotic Platforms in General Surgery: What’s the Current Clinical Scenario?

**DOI:** 10.3390/medicina59071264

**Published:** 2023-07-07

**Authors:** Francesco Marchegiani, Leandro Siragusa, Alizée Zadoroznyj, Vito Laterza, Orsalia Mangana, Carlo Alberto Schena, Michele Ammendola, Riccardo Memeo, Paolo Pietro Bianchi, Giuseppe Spinoglio, Paschalis Gavriilidis, Nicola de’Angelis

**Affiliations:** 1 Unit of Colorectal and Digestive Surgery, DIGEST Department, Beaujon University Hospital, AP-HP, University of Paris Cité, Clichy, 92110 Paris, France; marchegiani.fra@gmail.com (F.M.);; 2 Department of Surgical Sciences, University of Rome “Tor Vergata”, Viale Oxford 81, 00133 Rome, Italy; 3 Science of Health Department, Digestive Surgery Unit, University “Magna Graecia” Medical School, 88100 Catanzaro, Italy; 4 Unit of Hepato-Pancreato-Biliary Surgery, General Regional Hospital “F. Miulli”, 70021 Acquaviva delle Fonti, Italy; 5 Division of General and Robotic Surgery, Department of Health Sciences, San Paolo Hospital, University of Milan, 20142 Milan, Italy; 6 Research Institute Against Digestive Cancer (IRCAD), 67000 Strasbourg, France; 7 Department of Surgery, Saint Helena General Hospital, Jamestown, Saint Helena STHL 1ZZ, South Atlantic Ocean, UK

**Keywords:** robotic surgery, training, new surgical robots, environmental sustainability, colorectal surgery, hepatobiliary surgery, upper gastrointestinal surgery, abdominal wall surgery, endocrine surgery, breast surgery

## Abstract

*Background and Objectives*: Robotic surgery has been widely adopted in general surgery worldwide but access to this technology is still limited to a few hospitals. With the recent introduction of new robotic platforms, several studies reported the feasibility of different surgical procedures. The aim of this systematic review is to highlight the current clinical practice with the new robotic platforms in general surgery. *Materials and Methods*: A grey literature search was performed on the Internet to identify the available robotic systems. A PRISMA compliant systematic review was conducted for all English articles up to 10 February 2023 searching the following databases: MEDLINE, EMBASE, and Cochrane Library. Clinical outcomes, training process, operating surgeon background, cost-analysis, and specific registries were evaluated. *Results*: A total of 103 studies were included for qualitative synthesis after the full-text screening. Of the fifteen robotic platforms identified, only seven were adopted in a clinical environment. Out of 4053 patients, 2819 were operated on with a new robotic device. Hepatopancreatobiliary surgery specialty performed the majority of procedures, and the most performed procedure was cholecystectomy. Globally, 109 emergency surgeries were reported. Concerning the training process, only 45 papers reported the background of the operating surgeon, and only 28 papers described the training process on the surgical platform. Only one cost-analysis compared a new robot to the existing reference. Two manufacturers promoted a specific registry to collect clinical outcomes. *Conclusions*: This systematic review highlights the feasibility of most surgical procedures in general surgery using the new robotic platforms. Adoption of these new devices in general surgery is constantly growing with the extension of regulatory approvals. Standardization of the training process and the assessment of skills’ transferability is still lacking. Further studies are required to better understand the real clinical and economical benefit.

## 1. Introduction

Twenty-two years after the clinical introduction of the first Intuitive Surgical Da Vinci system, only a limited percentage of general surgery procedures are performed via robotic approach in Western countries [1]. In addition, there is a great disparity between developed and low-income countries where robotic surgery remains unsustainable despite its potential technical advantages [1,2].

Historically, AESOP^®^ and ZEUS, both produced by the American Computer Motion, were the first robotic surgical systems adopted in general surgery [3]. In 2003, after long legal action, American Computer Motion merged with its main competitor, Intuitive Surgical, which had been founded eight years prior [3]. The company developed several generations of master-slave multi-arm robots protecting their products thanks to the registration of more than 7000 patents which was the main barrier for the development of contenders [4,5]. After twenty years, the first registered patents are progressively expiring allowing the development of competing products [6]. The twenty years monopoly constituted an enormous advantage for the Intuitive Surgical company, whose products were adopted by most surgical specialties, thanks to the claimed technical advantages over laparoscopy provided by 3D imaging, magnification, dexterity, tremor filtration, motion scaling and a quick learning curve [5]. At the beginning of 2023, more than 11 millions robotic surgeries have been performed worldwide with Intuitive Surgical Da Vinci robots, with over 7500 platforms installed worldwide [7].

Nevertheless, the scenario is changing because new robotic platforms have been recently introduced into the market with several new architectures (e.g., modular platforms). Their use appeared to be feasible, but the associated surgical results and clinical effectiveness still require further investigation [8].

The aim of this systematic review was to evaluate the adoption of these new surgical robotic systems in general surgery in terms of clinical data, technical aspects, costs, and learning curve.

## 2. Materials and Methods

### 2.1. Search Strategy and Data Sources

The systematic review was performed according to the Cochrane Collaboration-specific protocol [9] and reported according to the Preferred Reporting Items for Systematic Reviews and Meta-Analyses (PRISMA) statement [10].

A first search was performed in grey literature and on the internet to identify the newly available robotic platforms, different from the Intuitive Surgical Da Vinci S^®^/Si^®^/Xi^®^/X^®^ (Intuitive Surgical, Sunnyvale, CA, USA). Studies describing the adoption of new robotic platforms in general surgery were searched in the following databases up to 10 February 2023: Medline (through PubMed), Embase, and Cochrane Library.

A specific research query was adopted for each database, using the following keywords: hugo robot; versius robot; da vinci single-port robot; flex robotic system robot; senhance robot; revo-i robot; microhand robot; hinotori robot; avatera robot; distalmotion robot; maestro robot; bitrack system robot; sport surgical system robot; mira robot; mantra robot. Due to the adoption of proper commercial names, multiple spellings for each word were adopted to avoid any missing data related to the improper name typing in the existing publications.

According to the PICOS format, the following items were used to select the retrieved articles:

P, population: patients > 18 years undergoing a robotic intervention with a platform different from Intuitive Surgical multiport Da Vinci S^®^/Si^®^/Xi^®^/X^®^.
I, intervention: any general surgery intervention with the following exclusions: gynecology, urology, thoracic surgery, othorinolaringoiatry, plastic surgery, pediatric surgery.
C, comparison: any comparison or no comparison.
O, outcomes: all reported outcomes, such as intraoperative, postoperative, short-term, long-term, functional, learning-curve, or cost analysis.
S, study design: due to the expected paucity of studies on the topic, all types of study design were considered, including case reports. Systematic and narrative reviews were excluded. Redundant studies were included and highlighted in the results. Abstract or congress communications were excluded. Only studies in English language were included.

The literature search and selection were performed by two independent reviewers (FM, LS). According to the PRISMA methodology, all records were first merged into a single database, then duplicates were removed, and the remaining articles were reviewed for relevance using the title and abstract. Disagreement was resolved by discussion and consensus; if no agreement was reached, a third senior author was consulted (NdA) in assessing study inclusion.

Finally, the two reviewers, supported by three supplemental reviewers (AZ, VL, OM) performed an independent full-text analysis to finalize the inclusion of pertinent articles.

The protocol has been registered in the International Prospective Register of Systematic Reviews database (PROSPERO: CRD42023416428).

### 2.2. Data Extraction and Synthesis

An electronic spreadsheet was filled with data extracted from the selected studies. The following items were collected: first author’s name, year of publication, country, type of study design, time frame of the study, pathological state requiring surgical intervention, number of patients/procedures evaluated, type of surgical intervention, adopted robotic platform, number of robotic and assistant arms adopted, number of surgeons involved, surgeon experience, surgical team experience, patient’s age, patient’s sex, intraoperative surgical outcomes, postoperative surgical outcomes, short-term outcomes, long-term outcomes, functional outcomes, learning-curve, or cost analysis.

### 2.3. Quality Assessment

The risk of bias of the included studies was assessed according to the MINORS scoring system. The MINORS system attributes a score of 0 if the item is not reported, 1 if the item is reported but inadequate, or 2 if the item is reported and adequate. The global highest score is 16 for non-comparative studies and 24 for comparative studies. Case reports were not evaluated due to the high risk of bias by definition.

## 3. Results

**Figure 1:**
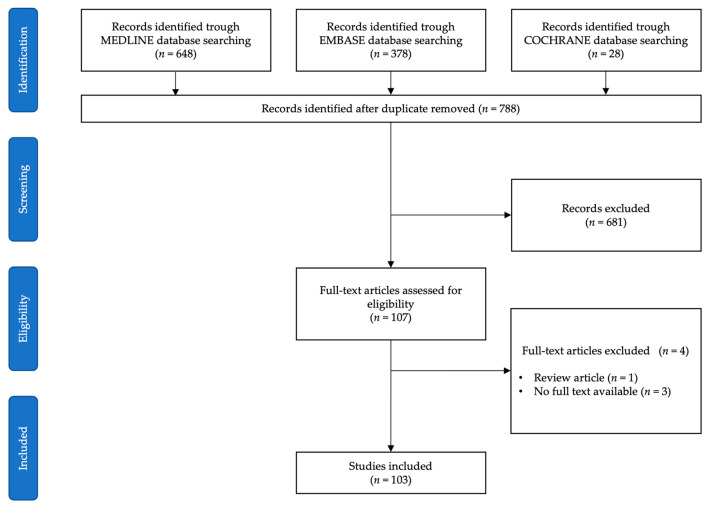
PRISMA flowchart of the literature search and selection.

The initial database search identified a total of 1054 studies, of which 266 were duplicates. After screening the titles and abstracts of the 788 remaining articles, 681 were excluded owing to non-pertinent specialty or intervention. After the full-text reading of the 107 eligible articles, a further 4 were excluded since 1 was a review article and 3 did not have a full-text version available. One-hundred and three studies met the inclusion criteria and were selected for the qualitative synthesis of the literature (Figure 1).

Among the included studies, 36 were case reports [11,12,13,14,15,16,17,18,19,20,21,22,23,24,25,26,27,28,29,30,31,32,33,34,35,36,37,38,39,40,41,42,43,44,45,46], 52 were noncomparative studies [47,48,49,50,51,52,53,54,55,56,57,58,59,60,61,62,63,64,65,66,67,68,69,70,71,72,73,74,75,76,77,78,79,80,81,82,83,84,85,86,87,88,89,90,91,92,93,94,95,96,97,98], and 15 were comparative studies [99,100,101,102,103,104,105,106,107,108,109,110,111,112,113]. Only one study was a randomized controlled trial [103].

The comparator was the Intuitive Surgical Da Vinci robot in ten cases [99,100,101,103,104,105,106,107,110,112] and the laparoscopic approach in six cases [102,105,106,108,109,111]. Only one study compared two different techniques with the same platform [113].

A total of 4053 patients were described, of whom 3099 were operated on with a new robotic platform. The population consisted of 1526 women (49.2%) and 1569 men (50.6%) and 4 patients (0.1%) whose sex was not specified. The age of the patients ranged between 15 and 92 years. Several series included the same population reducing the total number to 2819 patients (Table 1).

The authors belonged to institutions located in: South Korea (*n* = 27), China (*n* = 16), North America (*n* = 13), Lithuania (*n* = 11), Japan (*n* = 8), Germany (*n* = 6), Italy (*n* = 6), United Kingdom (*n* = 6), India (*n* = 4), United Arab Emirates (*n* = 2), Taiwan (*n* = 1), France (*n* = 1), Croatia (*n* = 1), Australia (*n* = 1).

The reported cases belonged to several specialties: hepatopancreatobiliary surgery [13,37,38,39,50,51,52,53,54,55,78,79,87,88,89,90,91,92,93,94,95,96,97,100,101,102,103] (Tables S1 and S2), colorectal surgery [14,15,16,17,18,19,20,21,22,23,24,25,26,27,28,29,30,31,32,33,34,35,36,56,57,58,59,60,61,62,63,64,65,66,67,68,69,70,71,72,73,74,75,88,89,90,91,93,94,95,96,97,98,104,105,106,107,108,109,110,111] (Tables S1 and S3), abdominal wall surgery [11,12,47,48,49,87,88,89,90,93,94,95,99] (Tables S1 and S4), endocrine surgery [76,77,81,82,83,112,113] (Table S5), upper gastrointestinal and bariatric surgery [42,43,44,45,71,84,86,88,95,96,97,98] (Tables S1 and S6), breast surgery [40,41,80] (Table S7).

The most performed procedure according to the specialty was: cholecystectomy in hepatobiliary surgery, anterior rectal resection in colorectal surgery, transabdominal pre-peritoneal hernia repair in abdominal wall surgery, transaxillar hemithyroidectomy in endocrine surgery, transthoracic esophagectomy in upper gastrointestinal surgery, and nipple sparing mastectomy in breast surgery.

Perioperative and postoperative outcomes were reported, respectively, in 101 (98.1%) [13,14,15,16,17,18,19,20,21,22,23,24,25,26,27,28,29,30,31,32,33,34,35,36,37,38,39,40,41,42,43,44,45,46,47,48,49,50,51,52,53,54,55,56,57,58,59,60,61,62,63,64,65,66,67,68,69,70,71,72,73,74,75,76,77,78,79,80,81,82,83,84,85,86,87,89,90,91,92,93,94,95,96,97,98,99,100,101,102,103,104,105,106,107,108,109,110,111,112,113] and 99 (96.1%) [13,14,15,16,17,18,21,22,23,24,25,26,27,28,29,30,31,32,33,34,35,36,37,38,39,40,41,42,43,44,45,46,47,48,49,50,51,52,53,54,55,56,57,58,59,60,61,62,63,64,65,66,67,68,69,70,71,72,73,74,75,76,77,78,79,80,81,82,83,84,85,86,87,88,89,90,91,92,93,94,95,96,97,98,99,100,101,102,103,104,105,106,107,108,109,110,111,112,113] studies. Five authors assessed the procedural learning curve [49,54,83,108,109]. One article investigated the patients’ satisfaction [53]. Functional results were reported in colorectal surgery by five authors only [104,105,106,108,109].

No major issues related to the robotic system were reported, except for five cases with the same robotic system, reported by three different authors, which did not generate consequent serious clinical events [48,49,50].

The mean MINORS score was 9 (4–14) and 16 (9–22) for non-comparative and comparative studies, respectively.

### 3.1. Surgery Setting

Most of the reported cases were elective surgeries (91.2%). Thirteen articles reported on emergency cases performed via a robotic approach. Two incarcerated hernias [12,88], one perforated gastric ulcer [96], seven acute appendicitis [91,96], and 99 acute cholecystitis [50,51,52,54,87,88,97,100] were described.

### 3.2. Robotic Platforms

Fifteen different robotic platforms were identified on the internet. Seven robotic platforms were identified as authorized for clinical use in at least one healthcare system (Medtronic Hugo^™^ RAS; Cambridge Medical Robotics Versius^®^; Intuitive Surgical Da Vinci SP^®^; Medrobotics Corp. Flex Robotic System; Asensus Senhance^®^ ALF-X; Meerecompany Inc. Revo-i^™^; Wego Micro Hand S) and their clinical results were reported. Five robotic systems have been authorized but no clinical data were available in scientific literature (Medicaroid Hinotori^™^; Avatera Medical Avatera^®^; Distalmotion Dexter; Moon Surgical Maestro; Virtual Incision MIRA). Three additional surgical platforms were detected but no clinical approval nor application was retrieved (Titan Medical Inc ENOS^™^; SS Innovation Mantra; Rob Surgical Systems S Bitrack System).

The regulatory approvals and the available information on these platforms are reported in Table 2.

#### 3.2.1. Patient Chart Architecture

Five systems (Medtronic Hugo^™^ RAS; Cambridge Medical Robotics Versius^®^; Asensus Senhance^®^ ALF-X; Distalmotion Dexter; SS Innovation Mantra) are modular with independent arms ranging from three to four, including the optical arm.

Five platforms (Meerecompany Inc. Revo-i^™^; Wego Micro Hand S; Medicaroid Hinotori^™^; Avatera Medical Avatera^®^; Rob Surgical Systems S Bitrack System) have a multiarm architecture with three to four arms, including the optical arm.

Two systems (Intuitive Surgical Da Vinci SP^®^; Titan Medical Inc ENOS^™^) are single port surgery platforms endowed with three to four arms, including the optical arm, characterized by flexible arms.

One robotic platform (Virtual Incision MIRA) shows a new miniaturized architecture allowing the entrance and the deployment of the two sterile arms and the optics directly into the body through a single incision.

One platform (Medrobotics Corp. Flex Robotic System) is a flexible endoscope with two operating arms.

One system (Moon Surgical Maestro) is intended to hold and position laparoscopes and laparoscopic instruments during laparoscopic surgical procedures, like an assisted laparoscopy rather than robotics (Table 3).

#### 3.2.2. Surgeon Console Architecture

The majority (64.3%) of the analyzed systems have an open console (Medtronic Hugo^™^ RAS; Cambridge Medical Robotics Versius^®^; Asensus Senhance^®^ ALF-X; Wego Micro Hand S; Virtual Incision MIRA; Titan Medical Inc. ENOS^™^; SS Innovation Mantra; Rob Surgical Systems S Bitrack System; Distalmotion Dexter). One of them (Distalmotion Dexter) does not have a dedicated viewing system but adopts a laparoscopic screen, considering that the surgeon console is sterile, and the operating surgeon is in the surgical field. 

Two robots have a closed console with a Da Vinci-like architecture (Intuitive Surgical Da Vinci SP^®^; Meerecompany Inc. Revo-i^™^).

Two systems adopt a semi-open console (Avatera Medical Avatera^®^; Medicaroid Hinotori^™^) with an immersive view into a closed viewer but without the bulky system, reducing the physical isolation of the operating surgeon.

One system (Medrobotics Corp. Flex^®^ Robotic System) does not have a real console but an open bidimensional screen to drive the endoscope and two mechanical arms directly controlled by the surgeon with no electromechanical mediation.

One system (Moon Surgical Maestro) is more a holder for laparoscope and instruments so it does not have a dedicated console (Table 3).

#### 3.2.3. Trocars, Instruments, and Reusability

Seven robotic platforms adopt commercial laparoscopic trocars (Cambridge Medical Robotics Versius^®^; Asensus Senhance^®^ ALF-X; Medtronic Hugo^™^ RAS; Meerecompany Inc. Revo-i^™^; Distalmotion Dexter; Moon Surgical Maestro; Rob Surgical Systems S Bitrack System), while three (Wego Micro Hand S; Medicaroid Hinotori^™^; SS Innovation Mantra) opt for a dedicated trocar. One system adopts a dedicated metallic trocar with a disposable commercial single site access system (Intuitive Surgical Da Vinci SP^®^)

Medrobotics Corp. Flex^®^ Robotic System is like a coloscope with no need for trocars.

Three platforms (Titan Medical Inc ENOS^™^; Virtual Incision MIRA; Avatera Medical Avatera^®^) did not still specify the adopted access system but two of them (Titan Medical Inc ENOS^™^; Virtual Incision MIRA) will probably opt for a single port commercial system.

All the described systems except for two (Asensus Senhance^®^ ALF-X; Moon Surgical Maestro) have wristed or flexible instruments. As an exception, some papers reported the adoption of wristed instruments (Radia^®^) for the the Asensus Senhance^®^ ALF-X [23,72,93]. Furthermore, the Wego Micro Hand S system is equipped by some authors with a rigid advanced ultrasonic dissector [69,84,96,103,106,109,110].

Nine platforms adopt reusable instruments (Intuitive Surgical Da Vinci SP^®^; Asensus Senhance^®^ ALF-X; Cambridge Medical Robotics Versius^®^; Meerecompany Inc. Revo-i^™^; Medicaroid Hinotori^™^; Wego Micro Hand S; Virtual Incision MIRA; Titan Medical Inc ENOS^™^; SS Innovation Mantra). One of them (Virtual Incision MIRA) is totally sterilizable and portable.

One system is partially sterilizable but adopts disposable instruments (Medrobotics Corp. Flex^®^ Robotic System).

Three robotic platforms use disposable instruments (Avatera Medical Avatera^®^; Distalmotion Dexter; Rob Surgical Systems S Bitrack System).

One robotic system (Medtronic Hugo^™^ RAS) uses sterilizable instruments with some disposable tools, as the needle driver and the scissor.

One system (Moon Surgical Maestro) does not have robotic tools so laparoscopic instruments can be adopted (Table 3).

#### 3.2.4. Advanced Energy and Staplers

All the platforms support monopolar and bipolar energy but only three systems (Asensus Senhance^®^ ALF-X; Wego Micro Hand S; Meerecompany Inc. Revo-i^™^) offer advanced ultrasonic energy.

A complete gamma of staplers or advanced energy is not currently available for any of the investigated platforms (Table 3).

### 3.3. Training

Forty-five (43.7%) authors reported the surgical background of the operating surgeon and his previous experience [26,31,33,45,47,48,49,50,51,52,53,54,55,56,57,58,59,62,64,67,69,76,77,79,83,84,85,86,87,88,89,90,91,92,94,96,99,102,103,104,105,106,108,109,110]. A structured training process of the surgical team on the adopted system was reported by 28 (27.2%) authors [26,40,47,48,49,51,52,53,54,56,58,59,60,64,67,76,77,85,87,88,90,91,92,93,95,99,109,110]. Of these, only five (4.8%) articles clearly stated the involvement of the nurses in the training process [54,64,77,93,99]. Only one (1%) article described the anesthesiologist as part of the team who underwent the surgical training process [93].

Concerning skill transferability, 26 (25.2%) articles reported a previous experience with a Da Vinci robot [26,31,33,45,47,53,54,56,62,67,69,77,79,83,85,87,88,89,90,96,99,103,104,105,106,110].

No article reported a previous experience with a platform different from Intuitive Surgical Da Vinci.

Three (2.9%) authors declared the existence of a credentialing program for the surgeon or the hospital [55,56,88]. Proctoring was mentioned by only six (5.8%) papers as part of the translational training during the first cases [48,56,87,88,93,99].

### 3.4. Registries

Two manufacturers (Asensus Surgical; Cambridge Medical Robotics) provided the surgeons with self-established registries whose results were published [94,124].

### 3.5. Costs

A cost analysis was performed by only one (1%) study [110]. The analysis compared the robotic total mesorectal excision performed with the Wego Micro Hand S or with its comparator benchmark, the Intuitive Surgical Da Vinci Si^®^. The Micro Hand S group had lower total hospital costs (87,040.1 ± 24,676.9 yuan vs. 125,292.3 ± 17,706.7 yuan, *p* < 0.05) and surgery costs (25,772.3 ± 4117.0 yuan vs. 46,940.9 ± 10,199.7 yuan, *p* < 0.05) when compared to the Da Vinci group.

## 4. Discussion

Robotic surgery has increasingly been adopted in general surgery since 2001 [125]. For years, the only widely adopted system was the Intuitive Surgical Da Vinci robot but, more recently, several other robotic platforms have been launched and introduced in the current practice after clinical approval in the respective markets. The present systematic review constitutes a state of the art of their clinical application.

The literature reviewed was very recent, published between 2016 and 2023, and reported clinical outcomes of over 2800 patients undergoing a minimally invasive operation with new robotic platforms.

Despite the recent adoption of these newly introduced platforms, the majority of the surgical procedures were performed with no reported adverse outcome and a low rate of technical issues related to the robot malfunction, confirming the reliability of the described systems. The new robots were mostly adopted for hepatopancreatobiliary, colorectal, and abdominal wall surgeries while fewer cases were reported for endocrine, upper gastrointestinal, and breast surgery. While some specialties, such as colorectal surgery, seemed to have extensively benefited from the new devices, others like the hepatopancreatobiliary surgery did not fully exploit their potential as the most performed procedure in such specialty still remains cholecystectomy. One of the reasons for this difference could be the absence of advanced instruments like staplers and powered dissectors, still not available for most of the presented robots. Another explanation could be found in the adoption of the new robotic platforms by hospitals aiming to improve the surgical volume and the attractiveness, even on simple procedures such as hernia surgery and cholecystectomy [126].

Nevertheless, the clinical indications for the new platforms are growing thanks to the constant approval of new specialties and new procedures in different countries.

The robotic platforms that are less represented in this review are expanding their market and new reports are available on a daily basis, following the acquisition by hospitals [127,128]. This robotic surgery broadening favored even general emergency surgeries for routine indications, such as appendicitis or cholecystitis, following a current trend in the literature [129]. Included articles belong to different continents but more than 50% were from Asia, where market is mainly driven by China, Japan, South Korea, India, and Taiwan.

These countries represent a population of more than 3 billion people, and they are pushing towards the development of indigenous platforms with the aim to compete with the existing Da Vinci. Concerning the Chinese market, the import of foreign robots is subject to a very limited quota, which was fully covered by Intuitive Surgical before the arrival of new competitors. The increasing complexity in the regulations will likely cut out expensive imported robots from the local market in favor of newly developed Chinese systems but it is not clear whether other countries will benefit from the commercialization of these platforms [130,131]. Access to the new technologies from China is extremely limited mainly due to the language barrier and the limited sharing of technical information, but currently several devices appear to be under testing and commercialization [132,133,134]. As for China, even Japan has a long history in robotic surgery, and it recently pushed the development of an indigenous product (Medicaroid Hinotori^™^). The company’s philosophy is tailored to suit the local market, promoting a smaller robot for smaller patients. However, it is also considering global expansion plans, as substantiated by its newly announced partnership with Karl Storz for the vision system [135] and the installation of the robot into a European training center [136]. Likewise, South Korea and India also developed their own systems with the aim of reducing robotic surgery expenses and facilitating the access to their population [137,138].

All the platforms presented in this review differ in nature, history, development, and technology. Only half of the included robots demonstrated their clinical potential while the rest are still under approval or in the investigational stage. The multi-arm robotic architecture invented by the Intuitive Surgical experience was adopted only by five manufacturers, whereas five others developed a modular concept more inspired by laparoscopy. Although the concept of “new robot” refers to small, portable, modular devices equipped with small instruments, this point was not agreed upon by the authors when reviewing the currently available robotic architecture, which is largely inspired by the well-known Da Vinci system. [139]. New concepts are emerging in the existing literature such as the mini-robot by Virtual Incision MIRA or the single port systems by Intuitive Surgical and Titan Medical emerged from the literature, leading to debates regarding indications and results, as it was in the laparoscopic era [140]. Additionally, two mentioned systems introduced the opportunity to robotize two routine practices such as the colonoscopy (Medrobotics Flex^®^) and the common laparoscopy (Moon Surgical Maestro). These innovations require proper trials to demonstrate their usefulness due to the current lack of clinical evidence.

Furthermore, analysis of instruments and trocars reveal differences across platforms in terms of materials, dimensions, and degree of articulation. Cost reduction, processability of instruments, tools’ precision, and CO_2_ emission remain top priorities for manufacturers despite the absence of common consensus, moreover regarding the use of reusable or disposable instruments.

These profound differences complicate a direct comparison between available robotic platforms. The only studies that aimed to show differences between manufacturers, were related to the Chinese Wego Micro Hand S system. The authors produced redundant literature demonstrating the equivalence of their new system with the existing Intuitive Surgical Da Vinci. In addition, they reported a decrease in hospital and surgical costs which could represent an advantage [110]. Additional economic studies are necessary to understand the real economic impact of the new platforms in various surgical environments.

The heterogeneous global situation, the variety of robots, and the continuous market growth are expected to revolutionize the clinical scenario in general surgery. Healthcare professionals will probably encounter multiple platforms throughout their career.

Due to the new paradigm of multiple robotic platforms possible co-existing in the same hospital, a proper credentialing system becomes essential.

The training process in surgery has been a topic of debate for decades, both in elective and emergency surgery [141]. Despite more than two decades of robotic surgery adoption as a surgical treatment across multiple specialties, no definitive training and credentialing programs were defined. Recently, Stefanidis et al. [142] and Burke et al. [143] tried to cope with this issue in the United States and United Kingdom, respectively. Currently, the proficiency assessment on the single platform is guaranteed by the manufacturer and hospitals grant permission to utilize the robot according to regulatory policies. This paper highlights that only 43.7% of the studies reported the operating surgeon’s previous experience. In addition, only 27.2% of the authors described the training process of the surgical team on the adopted platform. The involvement of the nurses and of the anesthesiologists is even more marginal, despite a growing interest in literature for the topic [144,145]. The proposed structured training from a single manufacturer confirms the technical ability of the surgeon to accomplish prefixed tasks in different settings (usually simulation, dry lab, wet lab on pigs and/or cadavers). Typically, the first clinical procedures performed are proctored, although this is reported by less than 6% of the authors.

These training modalities have been extensively adopted since the introduction of robotic surgery despite limited evidence and some conflict of interest. In fact, proficiency assessment is performed by the same company that has an interest in the robot’s clinical adoption. As there is no standardized curriculum in robotic surgery, analyzed papers did not provide information on the transferability of the skills from one platform to another. In a simulation environment, Larkins et al. were able to demonstrate some degree of robotic console skill transferability between two different multiport robotic platforms [146], while Ghazi et al. concluded for a partial transferability when simulating multiport and single-port robotic surgery [147]. In urology, currently the main market for robotic companies, a transition towards new systems were observed with positive clinical results [148], and it is reasonable to expect a similar process of validation for general surgery.

In order to validate the transition to new robotic devices, robotic companies are developing their products in collaboration with clinicians, trying to differentiate their approach and to collect data from the clinical activities. Two recently published registry analyses concerning the adoption of the two mainly diffused new generation robots, namely Asensus Senhance^®^ and CMR Versius^®^, reported 871 and 2083 cases, respectively [94,124]. The published databases will make the comparison of clinical outcomes simpler and more transparent. Furthermore, the registry promotion distinguished the competitors from Da Vinci.

The next stage of development will focus on the producer partnership to improve and ameliorate the existing products in order to better compete in a market still dominated by a single leader. The vision capabilities will be augmented thanks to the new technological standards, as announced in February 2023 by Asensus with its new Luna Surgical System endowed with a 4K-3D vision without the need to wear glasses.

In terms of clinical data analysis, the next major advancement will entail the adoption of artificial intelligence as proposed by Asensus or Medicaroid, in order to digitalize the surgical practice, opening the door to new opportunities such as the telesurgery [149,150].

The present review presents some limitations mainly related to the low-quality of existing evidence, the design and the small sample of studies included, and the absence of data on several robotic platforms. Nevertheless, this systematic review provides a good snapshot of the real clinical application of the recently introduced platforms in general surgery.

## 5. Conclusions

Robotic procedures with new robotic devices have been progressively described in hepatobiliary, colorectal, abdominal wall, upper gastrointestinal, endocrine and breast surgery. Despite the low-quality of the current evidence, this review suggests that most surgical interventions are feasible with no technical issues. More platforms are obtaining clinical approvals and their continuous development will be likely stimulated by the Asian market. However, the absence of an international training curriculum and credentialing program hinders the ability to evaluate surgical proficiency and the transferability of skills across different devices. Thus, the future holds substantial technological innovation whose clinical evidence is yet to be established.

## Figures and Tables

**Table 1:** Number of interventions according to the surgical specialties and the type of robotic platforms.

	Robotic Platform	Intuitive Surgical Da Vinci SP^®^	CMR Versius^®^	Asensus Senhance^®^	Wego MicroHand S/SII	Medrobotics Flex^®^	Meerecompany Revo-i^™^	Medtronic Hugo^™^ RAS	Total Cases Per Specialty
Surgical Specialty									
Hepatobiliary		386	422	114	96	0	17	0	1035
Colorectal		78	169	251	209	33	0	1	741
Abdominal wall		89	97	345	0	0	0	0	531
Endocrine		298	5	12	0	0	0	0	315
Upper GI		4	69	19	32	0	0	0	124
Breast		73	0	0	0	0	0	0	73
**Total cases per platform**		928	762	741	337	33	17	1	2819

**Table 2:** Regulatory approvals and available information of the robotic platforms.

Clinically Adopted Platforms				
Company	Product Name	Country	Regulatory Approvals	Marketing Information (n. Procedures/Platform)
Medtronic	Hugo^™^ RAS	US	FDA: ongoing CE-mark: general surgery; urology; gynecology Australian TGA: urology; gynecology Health Canada: general surgery MHLW PMDA Japan: urology; gynecology	NR
Cambridge Medical Robotics	Versius^®^	England	CE-mark: general surgery; urology; gynecology; thoracic surgery Australian TGA: general surgery; urology; gynecology Anvisa Brazil: general surgery; urology; gynecology Other countries: India; Pakistan; Egypt	10,000 procedures performed (March 2023) [114] >100 installed platforms (November 2022) [115]
Intuitive Surgical	Da Vinci SP^®^	US	FDA: urology; transoral procedures MHLW PMDA Japan: urology; gynecology; general surgery; thoracic surgery; transoral MFDS Korea: urology; general surgery; gynecology; thoracic surgery; transoral NMPA China: yes, not specified	121 installed platform (December 2022) [116] A’design award winner 2019
Medrobotics Corp.	Flex^®^ Robotic System	US	FDA: transoral; colorectal; general surgery; gynecology; thoracic surgery CE-mark: colorectal Australian TGA: colorectal	Bankrupt of the producing company
Asensus (formerly TransEnterix)	Senhance^®^ ALF-X	US	FDA: general surgery; gynecology. Pediatric surgery expected in 2023 CE-mark: general surgery; gynecology; pediatric surgery MHLW PMDA Japan: urology; gynecology; general surgery; thoracic surgery Roszdravnadzor—Russia: yes, not specified Taiwan: yes, not specified	>10,000 procedures performed (February 2023) >49 installed platforms between 2016 and 2022 [117]
Meerecompany Inc.	Revo-i^™^	South Korea	MFDS Korea: urology; gynecology; general surgery	NR
Wego	Micro Hand S	China	NMPA China: general surgery	Reddot award winner 2022
Platforms under Clinical Investigation				
Company	Product Name	Country	Regulatory Approvals	Marketing Information
Medicaroid	Hinotori^™^	Japan	MHLW PMDA Japan: urology; gastrointestinal; gynecology	840 procedures (December 2022) 28 installed platforms (September 2022) [118]
Avatera Medical	Avatera	Germany	CE-mark: urology; gynecology	Fist clinical procedure in May 2022 [119]
Distalmotion	Dexter	Switzerland	CE-mark: general surgery; gynecology	4 installed platforms [120] iF design award 2020
Moon Surgical	Maestro	US	FDA: laparoscopic procedures CE-mark: laparoscopic procedures	30 procedures performed [121]
Virtual Incision	MIRA	US	FDA: completed IDE for bowel resections. De novo classification pathway ongoing	NR
Titan Medical Inc.	ENOS^™^ (formerly SPORT)	Canada	FDA: planned in 2023 CE-mark: planned in 2023/24	NR
SS Innovation	Mantra	India	FDA: planned in 2023 CE-mark: planned in 2023 Other countries: India	5 installed platforms 100 procedures performed [122]
Rob Surgical Systems S	Bitrack System	Spain	NR	First clinical trial ongoing [123]

US: United States; FDA: food and drug administration; CE: Conformité Europeenne; TGA: Therapeutic Goods Administration; MHLW PMDA: Ministry of Health, Labour and Welfare Pharmaceuticals and Medical Devices Agency; NR: not reported; MFDS: Ministry of Food and Drug Safety; NMPA: National Medical Products Administration; IDE: Investigational Device Exemption.

**Table 3:** Summary of the overall characteristics of the robotic platforms.

Robotic Platform	Patient Chart Architecture	Console Architecture	Operative Arms No.	Trocars	Instruments	Instruments’ Reusability	Advanced Energy
Medtronic Hugo^™^ RAS	Modular	Open	3	Commercial	Wristed	Reusables (some disposables)	NA
Cambridge Medical Robotics Versius^®^	Modular	Open	3	Commercial	Wristed	Reusables	NA
Intuitive Surgical Da Vinci SP^®^	Single port	Closed	3	Dedicated + commercial	Wristed	Reusables	NA
Medrobotics Corp. Flex^®^ Robotic System	Flexible system	/	2	/	Wristed	Disposables	NA
Asensus Senhance^®^ ALF-X	Modular	Open	3	Commercial	Rigid with a kit of wristed	Reusables	Ultrasonic (rigid)
Meerecompany Inc. Revo-i^™^	Multiarm	Closed	3	Commercial	Wristed	Reusables	Ultrasonic (rigid)
Wego Micro Hand S	Multiarm	Open	2	Dedicated	Wristed	Reusables	Ultrasonic (rigid)
Medicaroid Hinotori^™^	Multiarm	Semi-open	3	Dedicated	Wristed	Reusables	NA
Avatera Medical Avatera	Multiarm	Semi-open	3	NR	Wristed	Disposables	NA
Distalmotion Dexter	Modular	Open (with laparoscopic screen)	2	Commercial	Wristed	Disposables	NA
Moon Surgical Maestro	Multiport instrument holder	/	1	Commercial	/	/	NA
Virtual Incision MIRA	Single port	Open	2	NR	Wristed	Reusables	NA
Titan Medical Inc. ENOS^™^ (formerly SPORT)	Single port	Open	2	NR	Wristed	Reusables	NA
SS Innovation Mantra	Modular	Open	3	Dedicated	Wristed	Reusables	NA
Rob Surgical Systems S Bitrack System	Multiarm	Open	3	Commercial	Wristed	Disposables	NA

NR: not reported; NA: not available.

## Data Availability

No new data were created or analyzed in this study. Data sharing is not applicable to this article.

## Supplementary Materials

The following supporting information can be downloaded at: https://www.mdpi.com/article/10.3390/medicina59071264/s1, Table S1: Mixed series; Table S2: Hepatopancreatobiliary surgery; Table S3: Colorectal surgery; Table S4: Abdominal wall surgery; Table S5: Endocrine surgery; Table S6: Upper gastrointestinal and bariatric surgery; Table S7: Breast surgery.
