# Supplementary material for: New Robotic Platforms in General Surgery: What’s the Current Clinical Scenario?
Source: Medicina (Kaunas). 2023 Jul 7;59(7):1264. doi: 10.3390/medicina59071264 (PMC10386395; doi:10.3390/medicina59071264)
Supplement: Supplementary file 1 [file medicina-59-01264-s001.zip › Table S1.pdf]

Table S1. Mixed series

References	Country	Study design	Time period	Surgical indication	No. of pts/ procedures	Type of intervention	Robotic platform	No. of operative arms	Surgeons involved	Surgeon experience	Surgical Team training	Main results
Case report/Technical notes												
Halabi H et al. (2022) [46]	United Arab Emirates	Case report	NS	Meckel diverticulum	1	Small bowel resection	CMR Versius®	3	2	NS	NS	Technical feasibility; no complications
Non-comparative studies												
Yi B. et al. (2016) [98]	China	Case series	mars 2014	1 gastric perforation; 2 acute appendicitis	3	1 gastric perforation repair; 2 appendectomies	Wego MicroHand S	2 (+ 1 AP)	NS	NS	NS	OT: 143.3 min; DT: 26.7 min; BL 41.7 ml; LOS: 4.3 days †
Yi B. et al. (2017) [97]	China	Case series	13 mos (Apr 2014 - Apr 2015)	1 gastric perforation; 3 acute appendicitis; 5 acute cholecystitis; 1 ileocaecal tumor	10	1 gastric perforation repair; 3 appendectomies; 5 cholecystectomies; 1 RC	Wego MicroHand S	2 (+ 1 AP)	1	NS	NS	Gastric perforation: OT: 145 min; DT: 40 min; BL: 25 ml; LOS: 5 days. Appendectomy: OT: 120 min; DT: 25 min; BL: 30 ml; LOS: 1 day. Cholecystectomy: OT: 130 min; DT: 30 min; BL: 35 ml; LOS: 1 day. RC: 160 min; DT: 25 min; BL: 50 ml; LOS: 5 days. No complications †
deBeche-Adams W. et al. (2019) [88]	US	Case series	3 mos (Apr 2018 - Jun 2018)	1 recurrent caecal adenoma; 1 caecal ulcer; 1 bilateral inguinal hernia; 1 incarcerated inguinal hernia; 1 gallstone pancreatitis; 1 chronic cholecystitis; 1 acute cholecystitis	7	3 cholecystectomies; 1 RC; 1 ileocaecal resection; 1 bilateral inguinal hernia repair; 1 right inguinal hernia repair	Asensus Senhance®	2 (± 1-2 AP)	3	Expert surgeons with years of experience in open, laparoscopy and robotics	Credentialing process: animal lab (at least 3 procedures/surgeon); proctoring during the first cases	RC and ileocecal resection LOS: 2 days. Cholecystectomies and hernia repairs LOS: 0 day (ambulatory) †
Montlouis-Calixte J. et al. (2019) [92]	France	Case series	6 mos (Jul 2017 - Dec 2017)	9 gynecological (6 adnexial pathologies, 2 endometriosis, 1 myoma); 5 cholelithiasis	14	9 gynecological (3 ovarectomies; 4 ovarian cystectomy; 1 myomectomy; 1 endometrial nodule resection); 5 cholecystectomies	Asensus Senhance®	2 (+ 1 AP)	3	Experienced laparoscopic surgeons	They received a specific training for the use of the Senhance® system	Cholecystectomy: OT: 87 min; conversion to laparoscopy: 7.1%; no complications †
Kelkar D. et al. (2020) [91]	India	Case series	1 month (Mar 2019 - Apr 2019)	9 cholelithiasis; 4 appendicitis; 17 gynecological diseases or infertility	30	9 cholecystectomies; 4 appendectomies; 17 gynecological procedures	CMR Versius®	2 (+ 1 AP)	6	High-volume, accredited surgeons	All members completed the validated 3.5-day Versius® training	OT: 120 min; no conversion; BL < 5 mL: 63.3%; BL < 500 mL: 36.7%; no Clavien–Dindo ≥III; 90-days readmission rate: 6.7%; LOS: 3 days
Samalavicius N.E. et al. (2020) [93]	Lithuania	Case series	5 mos (Nov 2018 - Mar 2019)	39 abdominal; 31 urologic; 30 gynecological	100	16 cholecystectomies; 15 colorectal resections; 8 TAPP; 31 urological procedures; 30 gynecological procedures	Asensus Senhance®	NS	2	NS	Surgeons, scrub nurses and anesthesiologists had a 2 day dry lab training and a 1 day wet lab	OT: 145 min; CR: 3%; Clavien–Dindo ≥III: 3%; no mortality

											on pigs, under proctor's guidance	
Yao Y. et al. (2020) [96]	China	Case series	57 mos (mars 2014 - Jan 2019)	sigmoid carcinoma; colon cancer; gastric cancer; rectal cancer; cholelithiasis; GIST; appendicitis; metabolic syndrome; choledochocyst; liver cancer; duodenal cancer; gastric ulcer; diverticulum of small intestine; pancreatic cancer	81	19 sigmoidectomies; 16 gastrectomies; 12 RC; 11 anterior resections; 5 left colectomies; 4 cholecystectomies; 3 gastric GIST resections; 3 appendectomies; 2 sleeve gastrectomies; 1 left lateral liver lobectomy; 1 choledochal cyst resection; 1 gastrojejunostomy; 1 partial gastrectomy; 1 diverticulectomy; 1 partial pancreatectomy	Wego MicroHand S	2 (+ 2 AP)	1	≥ 1 year of robotic surgery experience	NS	OT: 100–495 min; DT 7–54 min; BL: 20–1200 ml; LOS 4-30 days; CR: 3.7%; IO complications: 2.5%; no Clavien–Dindo ≥III; no readmission; no mortality †
Dixon F. et al. (2021) [89]	UK	Retrospective analysis of a prospectively collected database	19 mos (Nov 2019 - May 2021)	68 colorectal (54 malignant, 14 benign), 60 gynecologic (23 malignant, 37 benign), 32 general surgery (32 benign)	160	68 colorectal (22 RC; 25 anterior resections; 10 APR; 4 left colectomies; 7 others); 68 gyneacologic unspecified procedures; 32 general surgeries (18 inguinal hernias; 6 cholecystectomies; 8 ventral hernias)	CMR Versius®	NS	8	Consultants with experience in laparoscopy. Only one had experience in robotic surgery	NS	Colorectal: OT 170 min; conversion to open: 4.4%; Clavien–Dindo ≥III: 7.3%; LOS: 6 days; (3-34); readmission: 8.8%. General Surgery: OT 66 min; no conversion; LOS: 0 days; no Clavien–Dindo ≥III †
Stephan D. et al. (2021) [94]	Germany	Case series	41 mos (Feb 2017 - Jul 2020)	NS	871	220 unilateral hernia repairs; 70 bilateral hernia repairs; 159 cholecystectomies; 168 prostatectomies; 62 total hysterectomies; 192 others (visceral, colorectal, and gynecological)	Asensus Senhance®	NS	NS	Experienced laparoscopic surgeons	NS	DT: 7.5 min; OT: 114.3 min; conversion to laparoscopy: 3.7%; conversion to open: 1.6%; severe complications: 2.8% (20.8% related to the robot); no mortality
Bianco F. et al. (2022) [87]	US	Retrospective analysis of a prospectively collected database	26 mos (Jul 2019 - Sep 2021)	70 unilateral inguinal hernias; 7 bilateral inguinal hernias; 138 cholelithiasis or cholecystitis and cholelithiasis; 3 gallbladder polyps	217	77 TAPP; 141 cholecystectomy	Intuitive Surgical Da Vinci SP®	3	1	Robotic multiport and Da Vinci single site surgery experience	Training using simulation, 2 days of dry and wet lab, case observation, internal Urology	TAPP: OT 79.1 min; no conversions, hospital discharge: POD 0; no IO complications; no Clavien–Dindo ≥III; readmissions rate: 1.3%; port site incisional hernia: 1.3%. Cholecystectomy: OT 65.5 min; no conversions, hospital discharge: POD 0; no IO complications; no Clavien–Dindo ≥III; readmissions rate: 1.4%; port site incisional hernia: 1.4% †

											proctor for first cases	
Wehrmann S. et al. (2022) [95]	Germany	Retrospective analysis of a prospectively collected database	11 mos (Apr 2021 - Mar 2022)	NS	175	130 cholecystectomies; 27 colorectal (13 LARs; 11 RC; 3 appendectomies); 12 Upper GI (5 esophagectomies; 3 gastrectomies; 1 Heller myotomy; 1 fundoplication; 1 cardiomyotomy; 1 pyloroplasty); 5 HPB (2 liver cystectomy; 2 pancreatectomy; 1 left pancreatectomy); 1 splenectomy	CMR Versius®	3	2	NS	All the surgical team performed: online training, simulation, 3.5 day training programme with cadaveric model and animal model. The first cases were cholecystectomies	Cholecystectomy: OT: 82 min; BL: 10 ml; Clavien–Dindo ≥III: 0.8%; LOS: 3 days. Other procedures: OT range: 72-416; BL range: 0-800 ml; Clavien–Dindo ≥III: 8.9%; LOS range: 5-24 days †
El Dahdah J. et al. (2022) [90]	United Arab Emirates	Case series	3 mos (Oct 2021 - Dec 2021)	bilateral inguinal hernias; unilateral inguinal hernias; umbilical hernias; ventral hernias; appendix and gallbladder affections	55	30 hernia repairs; 22 cholecystectomies; 3 appendectomies	CMR Versius®	2 (± 1 AP)	3	>200/surgeon robotic Da Vinci surgery	Online course, 6h virtual training, 3 day dry lab	Bilateral inguinal hernia OT: 95.2 min; unilateral Inguinal hernia OT: 50.9 min; umbilical hernia: 71.3 min; ventral hernia: 115.5 min; cholecystectomy OT: 35.8 min; appendectomy OT: 28.3 min; no conversion; no complications †

All the reported values are absolute or median if not specified. † mean; NS: not specified; IO: intraoperative; PO: postoperative; LC: learning curve; OT: operative time; CT: console time; DT: docking time; CR: conversion rate; BL: blood loss; LOS: length of stay; AP: assistant port; RC: right colectomy
