# Supplementary material for: New Robotic Platforms in General Surgery: What’s the Current Clinical Scenario?
Source: Medicina (Kaunas). 2023 Jul 7;59(7):1264. doi: 10.3390/medicina59071264 (PMC10386395; doi:10.3390/medicina59071264)
Supplement: Supplementary file 1 [file medicina-59-01264-s001.zip › Table S2.pdf]

Table S2. Hepatopancreatobiliary surgery

References	Country	Study design	Time period	Surgical indication	No. of pts/ procedures	Type of intervention	Robotic platform	No. of operative arms	Surgeons involved	Surgeon previous experience	Surgical Team training	Main results
						Case report/Technical notes						
Cruz J.C. et al. (2019) [13]	South Korea	Case report	2019	Cholelithiasis	1	cholecystectomy	Intuitive Surgical Da Vinci SP®	3	1	NS	NS	OT: 89 min; DT: 6 min; no IO complications, no PO complications
Kang I. et al. (2020) [38]	South Korea	Case report	Dec 2018	Pancreatic insulinoma	1	PD (laparoscopic resection and robotic reconstruction)	Meerecompany Revo-i™	2 (+ AP)	NS	NS	NS	OT (laparoscopic + robotic): 514 min; BL: 200 ml; no complications; LOS: 10 days
Ku G. et al. (2020) [39]	South Korea	Case report	Dec 2019	Pancreatic NET	1	Central pancreatectomy (laparoscopic resection and robotic reconstruction)	Meerecompany Revo-i™	3 (+ AP)	2	NS	NS	OT: 295 min; BL: 50 ml; R0 resection; biochemical pancreatic fistula; LOS: 9 days
Kim W.-J. et al. (2021) [37]	South Korea	Case report	2020	Intrahepatic lithiasis	1	Left lateral sectionectomy	Intuitive Surgical Da Vinci SP®	3 (+1 AP)	NS	NS	NS	OT: 135 min; DT: 8 min; BL: 50 ml; no complications; LOS: 5 days †
						Non-comparative studies						
Melling N. et al. (2019) [54]	Germany	Retrospective analysis	4 mos (May 2017 - Aug 2017)	15 cholelithiasis; 3 chronic cholecystitis; 1 chronic cholecystitis and pancreatitis; 1 acute cholecystitis	20	Cholecystectomy	Asensus Senhance®	2 (+ 1 AP)	1	Previous laparoscopic and robotic Da Vinci experience	Surgeons and scrub nurse had 3 days of training course with simulation and pig lab activity	OT: 71.5 min; DT: 10 min; 1 conversion to laparoscopy: 5%; no complications; LC for DT after 10 cases
Aggarwal R. et al. (2020) [50]	UK	Retrospective analysis of a prospectively	NS	Cholelithiasis; cholecystitis; gallbladder polyp	20	Cholecystectomy	Asensus Senhance®	2 (3 robotic + 1	1	Over 1000 laparoscopic cholecystectomie	NS	OT: 86.5 (60.5-106.5); DT: 11.5 (9-13); IO complications: 2 (1 robot malfunction); no 30-days Clavien-Dindo≥3 complications †

		collected database						assistant)		s with only one conversion		
Lim J.H. et al. (2020) [53]	South Korea	Retrospective analysis	5 mos (Aug 2016 - Dec 2016)	9 chronic cholecystitis; 4 gallbladder polyps; 2 gallbladder polyps and cholelithiasis	15	Cholecystectomy	Meerecompany Revo-i™	3	1	>2000 laparoscopic cholecystectomies and >400 robotic Da Vinci cholecystectomies	12 hour specific Revo-i™ training was provided for the medical team	OT: 115.3 min; DT: 10.6 min; CT: 49.7 min; BL: 3.33 ml; no IO complications; no 30-days Clavien–Dindo ≥III; LOS: 2 days; 93.3% of patients would undergo another operation involving Revo-i™ †
Choi Y.J. et al (2022) [78]	South Korea	Case series	8 mos (Jun 2020 - Dec 2020)	1 pancreatic serous cystadenoma; 1 pancreatic NET; 1 pancreatic cancer	3	distal splenopancreatectomy	Intuitive Surgical Da Vinci SP®	3 (+1 AP)	1	NS	NS	OT: 215 min; DT 4.3 min; CT: 180.3 min; BL<500 ml; no complications; hospital discharge: POD 10.3 †
Liu R. et al. (2022) [79]	China	Retrospective analysis of a prospectively collected database	3 mos (Dec 2021 - Feb 2022)	resectable benign or borderline malignant pancreatic tumor	23	11 distal pancreatectomy; 11 pancreatic enucleation; 1 pancreaticoduodenectomy	Intuitive Surgical Da Vinci SP®	3 (+ 1/2 AP)	1	>1500 robotic Da Vinci pancreatic surgeries	NS	OT: 156.5 min; DT: 4.2 min; BL: 40 ml; no conversion; clinically relevant-PO pancreatic fistula: 13%; no Clavien-Dindo≥3; hospital discharge: POD 4; no 30-days readmission †
Khanna S. et al. (2022) [52]	India	Retrospective analysis of a prospectively collected database	18 mos (Feb 2020 - Aug 2021)	cholelithiasis; chronic and acute cholecystitis	106	Cholecystectomy; subtotal cholecystectomy; cholecystostomy	CMR Versius®	2 (3 BSU + 1 assistant trocar)	1	Over 40 years of biliary surgery experience	structured robotic training with virtual training, dry lab, wet lab and cadaver training in various procedures	CR 1.88% (1 conversion to laparoscopy; 1 conversion to open); no serious complications; overall CT: 54.16 min (6–205); overall setting up time: 9.29 min †
Sasaki T. et al. (2022) [55]	Japan	Retrospective analysis	19 mos (Sep 2020 - Mar 2022)	25 cholelithiasis; 5 chronic cholecystitis	30	Cholecystectomy	Asensus Senhance®	2 (+ 1 AP)	1	Certificated by the Japanese ESSQS of the JSES training program for SDLS	NS	OT: 69 min; DT: 4 min; CT: 34 min; BL: 1 ml; conversion to laparoscopy: 10%; no Clavien–Dindo ≥III; LOS: 3 days

Kelkar D.S. et al. (2023) [51]	India	Prospective	19 mos (Mar 2019 - Jul 2020)	114 symptomatic cholelithiasis; 26 cholecystitis; 3 polyps	143	Cholecystectomy	CMR Versius®	2 (+ 1 AP)	5	Accredited, practicing, high-volume, extensive experience in MIS. No device experience.	All surgical team members completed a didactic online program, a simulated practice and a 3.5 days training program	OT: 92 min; BL: 21.7% patients <5 ml; ,conversion to laparoscopic surgery 7/143 (4.8%), conversion to open: 1.4% (7 device related coneresions); Clavien–Dindo ≥III: 2.1%; mortality: 0.7%; LOS: 2 days; 30-days hospital readmission: 2.8%
Comparative studies - platforms												
Kang Y.H. et al. (2021) [100]	South Korea	Retrospective analysis	21 mos (Feb 2019 - Nov 2020)	cholelithiasis; polyps; adenomyomatosis; acute cholecystitis	330 (72 Da Vinci SP vs 258 Da Vinci Xi)	Cholecystectomy	Intuitive Surgical Da Vinci SP Vs Intuitive Surgical Da Vinci Xi with single-site system	3 vs 2 (+1 AP)	1	NS	NS	OT 45.9 min vs 43.4 min; CT 20.3 min vs 23.1 min (p = 0.018); BL: 19.2 ml vs 14.3 ml (p=0.031); complications: 0 vs 2; LOS: 2.3 days vs 1.1 days; readmissions: 0 vs 4 †
Samalavicius N.E. et al. (2021) [102]	Lithuania	Retrospective analysis	12 mos	cholelithiasis	40 (20 Senhance; 20 laparoscopic)	Cholecystectomy	Asensus Senhance® vs laparoscopy	NS	2	experienced surgeons	NS	OT 88.5 vs 60.8 min (p<0.05); DT 12 min; BL: 14.6 ml vs 11.3 ml; no conversion; Clavien–Dindo ≥III: 5% vs 0; LOS 1.5 days vs 1.55 days
Kim W-J. et al. (2022) [101]	South Korea	Retrospective analysis	50 mos (Mar 2017 - May 2021)	SP: 85 cholelithiasis; 24 polyp; 31 both; 5 adenomyoma. Xi: 92 cholelithiasis; 25 polyps; 40 both; 2 adenomyoma	304 (145 Da Vinci SP vs 159 Da Vinci Xi)	Cholecystectomy	Intuitive Surgical Da Vinci SP® Vs Intuitive Surgical Da Vinci Xi with single-site system	3 vs 2 (+1 AP)	1	NS	NS	OT: 45.7 min vs 49.8 min; DT: 5.7 min vs 8.8min (p=0.024); no conversion; Clavien–Dindo ≥III: 0.7% vs 0.6%; LOS 2.1 days vs 2.1 days
Wang G. et al. (2022) [103]	China	Multi-centre RCT	11 mos (Oct 2019 -	MicroHand: 82 chronic	168 (84 Micro Hand	Cholecystectomy	Wego MicroHand S vs Intuitive	2 (+1 AP) vs 2 (+1 AP)	2 / center	>5 years of endoscopic surgery, >1 year	NS	OT: 61 min vs 64 min; DT 12.0 min vs 16.4 (p=0.025); CT: 49.3 vs 48.6; BL 4.37 ml; 7.39 ml; gallbladder breach 4.8% vs 15.7% (p=0.021); no

			Sep 2020)	cholecystitis; 2 polyps. Da Vinci: 78 chronic cholecystitis; 6 polyps	vs 84 Da Vinci Si)		Surgical Da Vinci Si			of robotic surgery, both the surgeons passed the test product training		Clavien–Dindo ≥III in both groups; LOS 7 days vs 7 days †

All the reported values are absolute or median if not specified. † mean; NS: not specified; AP: assistant port; OT: operative time; DT: docking time; IO: intraoperative; PO: postoperative; LOS: length of stay; CT: console time; CR: conversion rate; BL: blood loss; NET: neuroendocrine tumor; PD: pancreaticoduodenectomy; LC: learning curve; MIS: minimally-invasive surgery; AP: assistant port.
