# Supplementary material for: New Robotic Platforms in General Surgery: What’s the Current Clinical Scenario?
Source: Medicina (Kaunas). 2023 Jul 7;59(7):1264. doi: 10.3390/medicina59071264 (PMC10386395; doi:10.3390/medicina59071264)
Supplement: Supplementary file 1 [file medicina-59-01264-s001.zip › Table S3.pdf]

**Table S3. Colorectal surgery**

| References | Country | Study design | Time period | Surgical indication | No. of pts/ procedures | Type of intervention | Robotic platform | No. of operative [illegible] | Surgeons involved | Surgeon previous experience | Surgical Team training | Main results |
|---|---|---|---|---|---|---|---|---|---|---|---|---|
| *Case report/Technical notes* | | | | | | | | | | | | |
| Samalavicius N.E. et al. (2019) [19] | Lithuania | Case report | 2019 | rectal adenoca | 1 | APR | Asensus Senhance® | 2 (+1 AP) | 1 | NS | NS | Technical feasibility; OT: 180 min; blood loss: 50 ml; no IO complications |
| Samalavicius N.E. et al. (2019) [20] | Lithuania | Case report | 2019 | right colon adenoca | 1 | RC | Asensus Senhance® | 2 | 1 | NS | NS | Technical feasibility; OT: 180 min; blood loss: 50 ml: no IO complications. |
| Samalavicius N.E. et al. (2019) [21] | Lithuania | Case report | 2019 | sigmoid colon adenoca | 1 | sigmoidectomy | Asensus Senhance® | 2 (+1 AP) | 1 | NS | NS | Technical feasibility; no PO complications. |
| Samalavicius N.E. et al. (2019) [22] | Lithuania | Case report | 2019 | rectal adenoca | 1 | hybrid taTME | Asensus Senhance® | NS | 1 | NS | NS | Technical feasibility; no PO complications; hospital discharge: POD7 |
| Arezzo A. et al. (2020) [32] | Italy | Case report | 2020 | rectal LST | 2 | full thickness excision; ESD | Medrobotics Flex® | 2 | NS | NS | NS | Case 1: OT: 55 min; no IO complications; no PO complications; hospital discharge: POD 1. Case 2: NS. |
| Hirano Y. et al. (2020) [18] | Japan | Case report | 2020 | sigmoid colon adenoca | 1 | LC | Asensus Senhance® | 2 (+1 AP) | 1 | NS | NS | OT: 204 min; blood loss: 75 mL; no IO complications; no PO complications; hospital discharge: POD 8 |
| Kataoka A. et al. (2020) [25] | Japan | Case report | 2020 | right colon adenoca | 1 | RC | Asensus Senhance® | 2/3 (dual docking) | 1 | NS | NS | OT: 313 min; DT: 18 min; blood loss: 5 ml; no IO complications; no PO complications; hospital discharge: POD 12 |
| Kondo H. et al. (2020) [26] | Japan | Case report | 2020 | cecal adenoca | 1 | ileocecal resection | Asensus Senhance® | 2 (+2 AP) | 1 | First robotic surgery experience | Dry lab | OT: 198 min; DT: 30-40 min; blood loss: 10 ml; no IO complications; No PO complications; hospital discharge: POD 9 |
| Marks J.H. et al. (2020) [33] | US | Case report | 2020 | cecal adenoca | 1 | RC | Intuitive Surgical Da Vinci SP® | 3 (+2 AP) | 1 | Extensive robotic experience | NS | Technical feasibility; OT: 219 min; DT: 19 min; blood loss: 100 ml; no IO complications; hospital discharge: POD 3 |

| | | | | | | | | | | | | |
|---|---|---|---|---|---|---|---|---|---|---|---|---|
| Marks J.H. et al. (2020) [31] | US | Case report | Oct 2018 | diverticular disease | 2 | LC | Intuitive Surgical Da Vinci SP® | 3 (+1 AP) | 1 | Cadaveric experience with the robot | NS | OT: 305 min; CT: 209 min; DT: 13; blood loss: 40 ml; no IO complications; no PO complications; LOS: 2.5 days † |
| Samalavicius N.E. et al. (2020) [21] | Lithuania | Technical note | 2019 | rectal adenoca | 1 | TME | Asensus Senhance® | 2 (+1 AP) | 1 | NS | NS | Technical feasibility; hospital discharge: POD 6 |
| Marks J.H. et al. (2021) [34] | US | Case report | 17 mos | rectal adedoma | 2 | full thickness excision | Intuitive Surgical Da Vinci SP® | 3 (+1 AP) | 1 | NS | NS | OT: 180.5 min; CT: 122.5; blood loss: 15 ml; no IO complications; LOS: 1 day † |
| Marks J.H. et al. (2021) [36] | US | Case report | 2 mos | rectal adedoma | 2 | taTME (only transanal time); fully taTME | Intuitive Surgical Da Vinci SP® | 3 for the transanal time | 1 | NS | NS | CT: 214.5 min; DT: 13.5 min; blood loss: 165 ml; no IO complications; no PO complications; hospital discharge: POD 2.5; 22.4 mos follow-up: no recurrence † |
| Marks J. et al. (2021) [35] | US | Technical note | NS | rectal adenoca | 1 | taTME | Intuitive Surgical Da Vinci SP® | 3 (+1 AP) | 1 | NS | NS | no IO complications; no conversion; blood loss: 50 ml; hospital discharge: POD 4; negative margins |
| Minagawa Y. et al. (2021) [16] | Japan | Case report | 2021 | transverse colon adenoca | 1 | single incision + 1 port transverse colectomy | Asensus Senhance® | 2 (+1 AP) | 1 | NS | NS | OT: 203 min; DT: 11 min; blood loss: 35 ml; PO anastomotic bleeding (Clavien-Dindo 3a); hospital discharge: POD 8. |
| Samalavicius N.E. et al. (2021) [23] | Lithuania | Technical note | 2021 | rectal NET | 1 | APR | Asensus Senhance® | 2 (+1 AP) | 1 | NS | NS | Technical feasibility; no PO complications; hospital discharge: POD7 |
| Alshalawi W. et al. (2022) [14] | South Korea | Technical note | 2022 | rectal adenoca | 1 | TME with ISR | Intuitive Surgical Da Vinci SP® | 3 (+1 AP) | 1 | NS | NS | OT: 185 min; estimated blood loss: 30 ml; hospital discharge: POD 6 |
| Bak M. R. et al. (2022) [15] | South Korea | Technical note | 2022 | rectal prolapse | 1 | ventral rectopexy | Intuitive Surgical Da Vinci SP® | 3 | 1 | NS | NS | Technical feasibility. No IO complications. No PO complications. |
| Cheong J.Y. Et al. (2022) [30] | South Korea | Technical note | 2022 | rectal adenoca | 1 | TME with ISR | Intuitive Surgical Da Vinci SP® | 3 (+1 AP) | 1 | NS | NS | DT: 7 min; CT: 180 min; no IO complications; blood loss: <50 ml; hospital discharge: POD 6 |
| Choo J.M. et al. (2022) [29] | South Korea | Technical note | 2022 | right colon adenoca | 1 | RC | Intuitive Surgical Da Vinci SP® | 3 (+1 AP) | 1 | NS | NS | DT: 4 min; CT: 134 min; no IO complications; hospital discharge: POD 4 |
| Piozzi G.N. et al. (2022) [28] | South Korea | Case report | 2022 | transverse colon adenoca | 1 | transverse colectomy | Intuitive Surgical Da Vinci SP® | 3 (+1 AP) | 1 | NS | NS | OT: 225 min; DT: 6 min; blood loss <50 ml; no IO complications; no PO complications; hospital discharge: POD 6; 17 mos follow-up: no recurrence |

| | | | | | | | | | | | |
|---|---|---|---|---|---|---|---|---|---|---|---|
| Campagna et al. (2023) [27] | Italy | Case report | 2023 | multicompartmental prolapse | 1 | sacrocolpopexy and ventral rectopexy | Medtronic Hugo™ RAS | 2 (+1 AP) | 1 | NS | NS | Technical feasibility; OT: 120 min; DT: 8 min; no IO complications; no PO complications; hospital discharge: POD 2 |
| Samalavicius N.E. et al. (2023) [24] | Lithuania | Technical note | 2022 | sigmoid colon adenoca | 1 | sigmoidectomy | Asensus Senhance® | 2 | 1 | NS | NS | Technical feasibility; no PO complications; hospital discharge: POD7 |
| ***Non-comparative studies*** | | | | | | | | | | | | |
| Spinelli A. et al. (2017) [64] | Italy | Retrospective analysis of a prospectively collected database | 16 mos (Jun 2015-Nov 2016) | 28 adenoca; 8 IBD; 5 diverticular disease; 2 NET; 2 adenoma | 45 | 23 RC; 12 AR; 9 LC; 1 total colectomy | Asensus Senhance® | 2/3 (+1/2 AP) | 1 | Extensive laparoscopic colorectal experience | The surgeon and the nurses received platform training | OT: 256 min; DT: 10.7; conversion to laparoscopy: 6.7%; blood loss: <50 ml; oncological R0 resection: 100%; Clavien-Dindo≥3: 4.4%; LOS: 5 days; hospital readmission: 2.2% |
| Collins D. et al. (2020) [56] | UK | Retrospective analysis of a prospectively collected database | 5 mos (Oct 2019 - Mar 2020) | adenoca | 32 | 18 RC; 14 AR | CMR Versius® | RC: 2 (+1 AP); TME: 3 (+1 AP) | 3 | Extensive laparoscopic colorectal experience. One surgeon had a robotic fellowship | 6h simulation; 32h dry lab and cadaver lab. 14h45 console. Proctoring for the first cases. | RC: OT: 221 min; CT: 111 min. AR: OT: 319 min; CT: 204 min. Blood loss: 150 ml; laparoscopic CR: 12.5%; open CR: 6.2%; no Clavien-Dindo≥3 PO complications; LOS: 5.3 days † |
| Darwich I. et al. (2020) [65] | Germany | Retrospective analysis | 12 mos (Oct 2017-Oct 2018) | 12 diverticular diseases | 12 | 12 sigmoidectomies | Asensus Senhance® | 2 (+1 AP) | 2 | NS | NS | OT: 219 min; CT: 149 min; DT: 10 min; blood loss: 20 ml; Clavien-Dindo≥3: 8.3%; LOS: 9 days † |
| Li J. et al. (2020) [69] | China | Retrospective analysis of a prospectively collected database | 10 mos (Mar 2018-Dec 2018) | adenoca | 25 | 25 RC with CME | Wego MicroHand S | 2 (+1 AP) | 1 | Laparoscopic CME since 2015, Da Vinci robotic CME since 2017, Micro Hand S CME since 2018 | NS | OT: 210 min; DT: 10 min; blood loss: 55 ml; no conversion; no Clavien-Dindo≥3; LOS: 7 days; no recurrence at 12-mos follow-up |

| | | | | | | | | | | | | |
|---|---|---|---|---|---|---|---|---|---|---|---|---|
| Lin C.-C. et al. (2020) [59] | Taiwan | Retrospective analysis of a prospectively collected database | 7 mos (Jun 2019-Dec 2019) | 39 adenoca; 2 diverticular disease; 3 adenoma; 2 rectal prolapse | 46 | 24 AR; 11 RC; 3 APR; 2 sigmoidectomy with rectopexy; 2 LC; 2 LAR; 1 SC; 1 TaTME | Asensus Senhance® | 2 (+1 AP) | 4 | First experience with the Senhance® platform | Senhance® robotic system training courses | DT: 25 min; CT: 112 min; OT: 283 min; blood loss: 50 ml; Clavien-Dindo≥3: 6.5%; mortality: 2.1%; LOS: 8 days † |
| Noh G.T. et al. (2020) [61] | South Korea | Retrospective analysis | 5 mos | 5 adenoca; 2 diverticular disease | 7 | 5 RC; 2 AR | Intuitive Surgical Da Vinci SP® | 3 (+2 AP) | 1 | NS | NS | OT: 300 min; DT: 20 min; CT: 225 min; blood loss: 50 ml; no IO complications; LOS: 7 days. |
| Salem J.F. et al. (2020) [62] | US | Retrospective analysis | NS | 4 diverticular disease | 4 | 4 LC | Intuitive Surgical Da Vinci SP® | 3 (+2 AP) | 1 | Involved in robotic development | NS | OT: 310 min; DT: 8.4 min; blood loss: 91 ml; no IO complications; LOS: 2.7 days † |
| Dixon F. et al. (2021) [57] | UK | Retrospective analysis of a prospectively collected database | 5 mos | 16 adenoca; 1 diverticular disease; 1 IBD; 5 benign (not specified) | 23 | 9 RC; 6 ARs; 4 APR; 1 LC; 1 Hartmann; 1 completion proctectomy; 1 proctocolectomy | CMR Versius® | NS | 2 | First experience in robotic surgery | NS | CT: 166 min; DT: 17 min; blood loss 100 ml; no IO complications; conversion to open: 4.3%; Clavien-Dindo≥3 PO complications: 8.7%; LOS: 5 days |
| Hirano Y. et al. (2021) [66] | Japan | Retrospective analysis of a prospectively collected database | 4 mos (Dec 2019-Mar 2020) | adenoca | 8 | 4 RC; 1 sigmoidectomy; 3 AR | Asensus Senhance® | 2 (+1 AP) | NS | NS | NS | OT: 229.1 min; CT: 139.1 min; blood loss: 49.4 ml; conversion to laparoscopy: 12.5% due to scope holder damage; no PO complications; LOS: 7.1 days |
| Kim. H.J. et al. (2021) [68] | South Korea | Retrospective analysis | 4 mos (Jul-Sep 2020) | rectal adenoca | 5 | 4 AR; 1 AR + ISR | Intuitive Surgical Da Vinci SP® | 3 (+2/3 AP) | 1 | NS | NS | OT: 195; CT: 130 min; DT: 4 min 20 sec; no open/laparoscopic conversion; blood loss: 15 ml; Clavien-Dindo≥3: 20%; LOS: 7 days |
| Marks J. et al. (2021) [75] | US | Prospective trial | 18 mos (Oct 2018 - Mar 2020) | 14 rectal adenomas; 10 rectal adenocas; 1 rectal GIST; 1 rectal carcinoid | 26 | 22 full thickness excision; 4 ESDs | Intuitive Surgical Da Vinci SP® | 3 (+1 AP) | 1 | NS | NS | OT: 124.2 min; DT: 6.4 min; R0 resection: Clavien-Dindo≥3: 3.8%; R0 resection rate: 100%; no recurrence at 5.9 mos follow-up † |

| | | | | | | | | | | | |
|---|---|---|---|---|---|---|---|---|---|---|---|
| Song S.H. et al. (2021) [73] | South Korea | Retrospective analysis | 4 mos (2020) | right colon adenoca | 5 | 5 RC | Intuitive Surgical Da Vinci SP® | 3 (+4 AP) | 1 | NS | NS | OT: 160 min; CT: 105 min; DT: 4 min 40 sec; blood loss: 20 ml; no IO complications; no conversion to open/laparoscopy; no Clavien-Dindo≥3; LOS: 7 days; no 30-days mortality |
| Fok K.Y. et al. (2022) [58] | Australia | Retrospective analysis | NS | 4 adenoma; 1 hyperplastic polyp | 5 | 4 ESD; 1 full thickness excision | Medrobotics Flex® | 2 | 1 | 9 y of experience with the TEM | 2-day cadaveric course training | OT: 143 min; no IO complications; no PO complications; LOS: 0.6 days † |
| Huscher C. et al. (2022) [67] | Italy | Retrospective analysis of a prospectively collected database | 7 mos (Jul 2020-Dec 2020) | 6 adenocas | 6 | 3 RC; 2 sigmoidectomies; 1 AR | CMR Versius® | 3 (+1 AP) | 2 | >1500 surgeries with multiple robots | The surgeons completed the CMR surgical-validated training (virtual simulation, cadaveric training). CMR team proctoring. | For RC: OT: 160 min. For sigmoidectomies: 246 min. For AR: 250 min. No IO complications; blood loss: 1 patient 100-500 ml; no Clavien-Dindo≥3 complications; LOS: 6.5 days |
| Liu S. et al. (2022) [74] | US | Retrospective analysis | NS | 4 rectal adenomas; 1 rectal adenoca | 5 | full thickness excision | Intuitive Surgical Da Vinci SP® | 3 (+1 AP) | NS | NS | NS | no IO complications; 1 readmission |
| Morino M. et al. (2022) [60] | Italy | Retrospective analysis of a prospectively collected database | 16 mos (Oct 2018 - Feb 2020) | 22 adenoma; 4 adenoca | 26 | 14 full thickness excision; 12 ESD | Medrobotics Flex® | 2 | NS | NS | 2-day training (dry lab, wet lab, cadavers), completing a full procedure (dissection and suturing) | OT: 115 min; 6 conversions to TEO; 1 unintentional IO perforation; R1 resection: 15%; 30-days Clavien-Dindo≥3: 3.8%; LOS: 3 days; 12 mos follow-up: 11.5% recurrence |
| Piozzi G.N. et al. (2022) [70] | South Korea | Retrospective analysis of a prospectively collected database | 13 mos (Nov 2020-Dec 2021) | 6 adenocas; 1 squamous cell carcinoma | 13 | 7 ISR; 5 RC; 1 transverse colectomy | Intuitive Surgical Da Vinci SP® | 3 (+2 AP) | 1 | NS | NS | For ISR: OT: 280 min; DT: 7 min; CT: 157 min. For right/transverse colectomies: OT: 220 min; DT: 5 min; CT: 155 min. Blood loss: <50 ml; no IO complications; no conversion; Clavien-Dindo≥3: 14.3%; LOS: 7 days; 2 systemic recurrence at 8 mos follow-up |
| Puntambekar S.P. et al. (2022) [71] | India | Retrospective analysis of a prospectively | 8 mos (Aug 2019- | adenoca | 31 | 31 ARs with TME | CMR Versius® | 2 (+3 AP) | NS | NS | NS | CT: 51 min; DT; 17 min; no conversion; blood loss: 55 ml; no Clavien-Dindo≥3 † |

| | | | | | | | | | | | | |
|---|---|---|---|---|---|---|---|---|---|---|---|---|
| | | collected database | Mar 2020) | | | | | | | | | |
| Samalavicius N.E. et al. (2022) [72] | Lithuania | Retrospective analysis of a prospectively collected database | 24 mos (Nov 2018-Nov 2020) | 48 adenoca; 9 adenoma; 1 FAP; 1 diverticular disease | 57 | 16 RC; 11 ARs with PME; 10 sigmoidectomies; 9 ARs with TME; 5 APR; 4 taTME; 1 SC; 1 AR with PME and Hartmann | Asensus Senhance® | 2 (+1 AP) | NS | NS | NS | OT: 194 min; blood loss: 20 ml; conversion to open: 3.4%; Clavien-Dindo≥3: 7%; LOS: 8 days † |
| Sasaki M. et al. (2022) [63] | Japan | Retrospective analysis | 36 mos (May 2018-May 2021) | adenoca | 55 | 18 ileocecal resection; 8 RC; 1 transverse colectomy; 9 sigmoid colectomy; 18 AR; 1 ISR | Asensus Senhance® | 2 (+1/2 AP) | NS | NS | NS | OT: 240 min; DT: 8 min; CT: 126 min; blood loss: 5 ml; no IO complications; LOS: 7 days |
| *Comparative studies - platforms* | | | | | | | | | | | | |
| Luo D. et al. (2020) [107] | China | Retrospective comparative study | 24 mos (Jan 2017-Jan 2019) | sigmoid adenoca | 45 (21 MicroHand S; 24 Da Vinci) | 45 sigmoidectomies | Wego MicroHand S; Intuitive Surgical Da Vinci (NS) | NS | NS | NS | NS | MicroHand S: OT: NS; conversion to open: 4.8%; blood loss: 118.6 ml; LOS: 9.1 days. Da Vinci: OT: 241.7 min; conversion to open: 8.3%; blood loss: 256.7 ml; LOS: 9.8 days † |
| Zeng Y. et al. (2020) [111] | China | Retrospective comparative study | 14 mos (Jan 2018 - Feb 2019) | right colon adenoca | 22 (10 MicroHand S; 12 laparoscopic) | 22 RC | Wego MicroHand S | 2 (+2 AP) | NS | NS | NS | For the MicroHand S: lower CCI: 11.7 ($p<0.05$); shorter hospital discharge: 11.4 days ($p<0.05$) † |
| Jiang J. et al. (2021) [104] | China | Prospective comparative study | 19 mos (May 2017-Dec 2018) | rectal adenoca | 90 (47 Da Vinci Si; 43 MicroHand S) | For the MicroHand S: 42 AR with TME; 1 APR | Wego MicroHand S; Intuitive Surgical Da Vinci Si | 2 (+1 AP) | 1 | Laparoscopic TME since 2015, Da Vinci robotic TME since 2016 and Micro Hand S robotic TME since 2017 | NS | For the MicroHand S: OT: 250 min; DT: 23 min; CR: 2.3%; blood loss: 57 ml; Clavien-Dindo≥3: 9.3%; LOS: 7 days; no difference in any outcomes between Da Vinci Si and MicroHand S; no difference in F outcomes |

| | | | | | | | | | | | |
|---|---|---|---|---|---|---|---|---|---|---|---|
| Lei Y. et al. (2021) [105] | China | Prospective comparative study | 28 mos (Jan 2017-May 2019) | rectal adenoca | 134 (46 laparoscopic; 45 Da Vinci Si; 43 MicroHand S) | For the MicroHand S: 42 AR with TME; 1 APR | Wego MicroHand S; Intuitive Surgical Da Vinci Si | Wego MicroHand S: 2 (+1 AP); Intuitive Surgical Da Vinci Si: 3 (+1 AP) | 1 | Performed 200 laparoscopic TME, 30 robotic TME using Da Vinci Si and 55 TME using MicroHand S | NS | For the MicroHand S: OT: 235 min; docking: 20.1 min (significantly longer compared to Da Vinci); CR: 2.3%; Clavien-Dindo≥3: 9.3%; LOS: 7.4 days; F recovery was faster in the robotic surgery group but recovered to preoperative level in all the three groups at 12 mos † |
| Wang Y. et al. (2021) [109] | China | Retrospective comparative study | 40 mos (Jul 2015-Nov 2018) | rectal adenoca | 105 (65 laparoscopic; 40 MicroHand S) | For the MicroHand S: 38 AR with TME; 2 APR | Wego MicroHand S | 2 (+2 AP) | 1 | Performed 300 open and >100 laparoscopic colorectal surgeries. Assisted >50 laparoscopic cases. | Training with MicroHand S | For the MicroHand S: OT: 339.3 min; blood loss: 140.9 ml; conversion to open: 2.5%; Clavien-Dindo≥3: 7.5%; LOS: 9.2 days; no F difference between robotic and laparoscopic TME; LC: after 17 cases significative reduction in OT † |
| Zeng Y. et al. (2021) [110] | China | Retrospective comparative study with PSM | 58 mos (Jan 2016-Oct 2020) | rectal adenoca | 54 (39 Da Vinci; 15 MicroHand S) - 28 considered for the PSM | 54 AR with TME | Wego MicroHand S; Intuitive Surgical Da Vinci Si | 2 (+2 AP) | 2 | >15 y laparoscopic surgery and >5 y with Da Vinci robot. Involved in the MicroHand development and >100 surgeries. | NS | For the MicroHand S: OT: 260.6 min; CT: 143.3 min; DT: 24.2 min (vs 17.1 min of Da Vinci, p=0.021 at PSM); LOS: 13.2 days; total hospital costs 87,040.1 yuan (vs 125,292.3 of the Da Vinci, p<0.05 at PSM); surgery costs: 25,772.3 yuan (vs 46,940.9 yuan of the Da Vinci, p<0.05 at PSM) † |
| Liu Y. et al. (2022) [106] | China | Prospective comparative study | 36 mos (Jan 2017-Jan 2020) | rectal adenoca | 135 (45 laparoscopic; 47 Da Vinci Si; 43 MicroHand S) | For the MicroHand S: 42 AR with TME; 1 APR | Wego MicroHand S; Intuitive Surgical Da Vinci Si | NS | 1 | 200 laparoscopic TME; 30 Da Vinci robotic TME; 35 TME using MicroHand S | NS | For the MicroHand S: OT: 235 min; blood loss: 66.6 ml; CR: 2.3%; Clavien-Dindo≥3: 7%; F recovery was faster in the robotic surgery group but recovered to preoperative level in all the three groups at 12 mos. No F difference between the robotic platforms † |

| | | | | | | | | | | | | |
|---|---|---|---|---|---|---|---|---|---|---|---|---|
| Wang Y. et al. (2022) [108] | China | Retrospective comparative study | 12 mos (May 2017-Apr 2018) | rectal adenoca | 99 (29 open; 44 laparoscopic; 26 MicroHand S) | For the MicroHand S: 25 AR with TME; 1 APR | Wego MicroHand S | 2 (+1 AP) | 1 | 5 y open surgery; 2 y laparoscopic surgery; simple robotic procedures; first experience with robotic TME | NS | For the MicroHand S: OT: 295.4 min; conversion to open: 3.8%; blood loss: 99.2 ml; Clavien-Dindo≥3: 7.7%; LOS: 8.9 days; surgical success: 84.6%; no F difference between robotic, open and laparoscopic TME; LC: after 13 cases significative reduction in OT † |

All the reported values are absolute or median if not specified. † mean; NS: not specified; IO: intraoperative; PO: postoperative; LC: learning curve; OT: operative time; CT: console time; DT: docking time; CR: conversion rate; LOS: length of stay; AP: assistant port; NET: neuroendocrine tumor; Adenoca: adenocarcinoma; RC: right colectomy; AR: anterior resection; LC: left colectomy; APR: abdominoperineal resection; SC: subtotal colectomy; ISR: intersphincteric resection; ST: short-term; LT long-term; F: functional; CA: cost analysis
