# Supplementary material for: New Robotic Platforms in General Surgery: What’s the Current Clinical Scenario?
Source: Medicina (Kaunas). 2023 Jul 7;59(7):1264. doi: 10.3390/medicina59071264 (PMC10386395; doi:10.3390/medicina59071264)
Supplement: Supplementary file 1 [file medicina-59-01264-s001.zip › Table S4.pdf]

Table S4. Abdominal wall surgery

References	Country	Study design	Time period	Surgical indication	No. of pts/ procedures	Type of intervention	Robotic platform	No. of operative arms	Surgeons involved	Surgeon previous experience	Surgical Team training	Main results
Case report/Technical notes												
Kim C.H. et al. (2022) [11]	South Korea	Technical note	2022	umbilical hernia	1	eTEP ventral hernia repair	Intuitive Surgical Da Vinci SP®	2	1	NS	NS	Technical feasibility
Kim D. et al. (2022) [12]	South Korea	Technical note	2022	incarcerated inguinal hernia	1	unilateral SP-TEP	Intuitive Surgical DaVinci SP®	2	1	NS	NS	Technical feasibility
Non-comparative studies												
Schmitz R. et al. (2019) [49]	Germany	Retrospective analysis	7 mos (Mar 2017 - Sep 2017	inguinal hernias	64 (52 unilateral; 12 bilateral)	unilateral and bilateral MP-TAPP	Asensus Senhance®	2	3	Experience with TAPP	Simulation, observation of live cases and animal lab	OT: 48 min; DT: 7 min; conversion to laparoscopy: 3.1% (1 robot malfunction); no complications; LOS: 1 day; no LC detected
Dixon F. et al. (2022) [47]	UK	Case series	12 mos (NS)	inguinal and ventral hernias	41 (28 unilateral; 4 bilateral; 9 ventral hernias)	unilateral and bilateral MP-TAPP	CMR Versius®	2	NS	Extensive experience in MIS, but only one had previous robotic experience	Dual consultant operations. CMR surgical-validated training program (simulation, cadavers)	Inguinal hernia CT: 41 min (13-141); ventral hernia CT: 26 min (8-56); inguinal hernia DT: 8 (5-18) min; ventral hernia DT: 13 (5-25) min; Inguinal hernia CR: 9,4%; Ventral hernia CR: 11,1%; no Clavien-Dindo≥3; no PO readmission
Samalavicius N.E. et al. (2022) [48]	Lithuania Germany Belarus	Registry analysis	36 mos (Mar 2017 – Mar 2020)	inguinal and ventral hernias	271 (203 unilateral; 68 bilateral)	unilateral and bilateral MP-TAPP; IPOM ventral hernia repair	Asensus Senhance®	2	NS	Experienced surgeons	Training center. Proctor for the first surgeries	OT for unilateral 74 ± 35 (32–265) and OT for bilateral 104 ± 36 (60–220); DT: 7±3 (1–90); CR: 4.4% (8 conversion to laparoscopy; 4 conversion to open); 5 IO complications (3 robot malfunction); Clavien-Dindo IIIb rate: 1.85% †
Comparative studies - platforms												

Dreifuss N.H. et al. (2022) [99]	US	Retrospective comparative study of a prospectively collected database	116 mos (Jul 2012 - Mar 2022)	inguinal hernias	465 (304 unilateral MP-TAPP; 74 bilateral MP-TAPP; 79 unilateral SP-TAPP; 8 bilateral SP-TAPP)	unilateral and bilateral MP-TAPP vs unilateral and bilateral SP-TAPP	Intuitive Surgical Da Vinci SP®; Intuitive Surgical Da Vinci Si	NS (MP) vs 2 (SP)	1	10-years experience in TAPP, robotic multiport, and Da Vinci single-site surgery.	Simulation, dry/wet lab, and case observation. Proctoring for the first 3 cases. Nursing staff, scrub techs and residents were trained.	OT SP-TAPP: 78.1 (26-149) min; DT SP-TAPP: 2.2 (1-10) min; SP-TAPP OT < MP-TAPP in unilateral hernias (p=0.003); no IO complications; no conversion; same day discharge: SP-TAPP > MP-TAPP (p=0.001). Recovery time: SP-TAPP < MP-TAPP (p<0.001). LOS: SP-TAPP < MP-TAPP (p<0.001); no serious PO complications; 30-day mortality and readmission rate were similar. Chronic pain, hernia recurrence, and port-site incisional hernias rates were similar †

All the reported values are absolute or median if not specified. † mean; NS: not specified; IO: intraoperative; PO: postoperative; LC: learning curve; OT: operative time; CT: console time; DT: docking time; CR: conversion rate; LOS: length of stay; TEP: totally extraperitoneal; TAPP: transabdominal pre-peritoneal; MP: multi-port; SP: single-port; IPOM: intraperitoneal onlay mesh; MIS: minimally-invasive surgery; AP: assistant port.
