# Supplementary material for: New Robotic Platforms in General Surgery: What’s the Current Clinical Scenario?
Source: Medicina (Kaunas). 2023 Jul 7;59(7):1264. doi: 10.3390/medicina59071264 (PMC10386395; doi:10.3390/medicina59071264)
Supplement: Supplementary file 1 [file medicina-59-01264-s001.zip › Table S5.pdf]

Table S5. Endocrine surgery

References	Country	Study design	Time period	Surgical indication	No. of pts/ procedures	Type of intervention	Robotic platform	No. of operative	Surgeons	Surgeon previous experience	Surgical Team training	Main results
Non-comparative studies												
Kim K. et al. (2020) [82]	South Korea	Case series	5 mos (Dec 2018 - Apr 2019)	papillary thyroid carcinoma	10	Transaxillary hemithyroidectomy	Intuitive Surgical Da Vinci SP®	3 (+ 1 AP)	2	NS	NS	OT: 148 min, DT: 4.9 min; CT: 80.7 min; no complications; hospital discharge: POD 3 †
Knezevic N. et al. (2022) [76]	Croatia	Case series	10 mos (May 2019 - Mar 2020)	11 adrenal adenoma; 1 benign adrenal cyst	12	Transabdominal adrenalectomy (7 left; 5 right)	Asensus Senhance®	2 (+ 1 AP for right side)	1	High-volume, extensive laparoscopic adrenalectomy experience	Well-trained and experienced assistants, all received specific training on the robotic system	OT: 165.1 min; DT: 11.6 min; CT: 98.6 min; conversion to laparoscopy: 8.3%; BL: 47 ml; Clavien-Dindo≥3: 8.3%; LOS: 4.5 days †
Park J. et al. (2022) [83]	South Korea	Retrospective analysis	7 month (Oct 2021 - Apr 2022)	42 papillary thyroid carcinoma; 4 adenomas; 2 NIFTP; 1 follicular thyroid cancer; 1 poorly differentiated cancer	50	Transaxillary hemithyroidectomy	Intuitive Surgical Da Vinci SP®	3	1	Extensive experience in transaxillary robotic thyroidectomy (>500 procedures)	NS	OT: 57.8 min; DT: 2.4min; CT: 25.5 min; Clavien-Dindo≥3: 2%; LC for OT: 20 cases †
Raffaelli M et al. (2022) [77]	Italy	Case series	Jul 2022	3 Cushing's syndromes; 1 adrenal cystic lesion; 1 pheochromocytoma	5	Transabdominal adrenalectomy (3 left; 2 right)	Medtronic Hugo™ RAS	2 (+ 1 AP)	1	Experienced in both laparoscopic and robotic adrenalectomy	All participating surgeons and nurses completed the technical training delivered by the company at a training center	OT: 119 min; DT: 5 min; CT: 55 min; no Clavien-Dindo≥3; LOS: 2 days
Ho J. et al. (2023) [81]	South Korea	Retrospective analysis	19 mos (Jan 2020 - Jul 2021)	papillary thyroid carcinoma	30	Transaxillary hemithyroidectomy + lateral neck dissection	Intuitive Surgical Da Vinci SP®	3 (+ 1 AP)	1	NS	NS	OT: 293.8 min; CT: 191.8 min; no Clavien-Dindo≥3; LOS: 4.8 days †

Comparative studies												
Kim J.K. et al. (2022) [113]	South Korea	Retrospective analysis	21 month (Jan 2019 - Sep 2021)	190 papillary thyroid carcinoma; 10 benign nodules	200	36 conventional skin flap vs 164 two-step retraction technique (177 transaxillary hemithyroidectomy; 23 transaxillary thyroidectomy)	Intuitive Surgical Da Vinci SP®	3 (+ 1 AP)	2	NS	NS	Conventional skin flap OT: 115.3 min; DT: 5.2 min; CT: 48 min; no Clavien-Dindo≥3. Two-step method OT: 116.7 min; DT: 3.8 min; CT: 60 min; no Clavien-Dindo≥3 †
Lee I.A. et al (2022) [112]	South Korea	Retrospective analysis	17 mos (Feb 2020 - Jun 2021)	6 hyperaldosteronism; 1 adenoma; 1 incidentaloma vs 7 hyperaldosteronism; 2 pheochromocytoma, 1 Cushing; 1 incidentaloma	19 (8 Da Vinci SP vs 11 Da Vinci Si/Xi)	1 transabdominal adrenalectomy; 7 retroperitoneal adrenalectomy vs 11 retroperitoneal adrenalectomy	Intuitive Surgical Da Vinci SP® Vs Intuitive Surgical Da Vinci Si/Xi with single-site system	3 (+ 1 AP) vs NS	1 vs NS	NS	NS	OT: 99.0 min vs 121.9 min; DT: 4.8 vs 7.7; conversion to laparoscopy: 0 vs 18.2%; BL: 2.5 ml vs 17.3 ml; no complications; LOS: 2.5 days vs 3.5 days †

All the reported values are absolute or median if not specified. † mean; NS: not specified; IO: intraoperative; PO: postoperative; LC: learning curve; OT: operative time; CT: console time; DT: docking time; CR: conversion rate; BLO: blood loss; LOS: length of stay; AP: assistant port; ST: short-term; LT long-term; NIFTP: non-invasive follicular thyroid neoplasm with papillary-like nuclear feature.
