# Supplementary material for: New Robotic Platforms in General Surgery: What’s the Current Clinical Scenario?
Source: Medicina (Kaunas). 2023 Jul 7;59(7):1264. doi: 10.3390/medicina59071264 (PMC10386395; doi:10.3390/medicina59071264)
Supplement: Supplementary file 1 [file medicina-59-01264-s001.zip › Table S6.pdf]

Table S6. Upper gastrointestinal and bariatric surgery

References	Country	Study design	Time period	Surgical indication	No. of pts/ [illegible]	Type of intervention	Robotic platform	No. of operative arms	Surgeons [illegible]	Surgeon previous experience	Surgical Team training	Main results
Case report/Technical notes												
Dreifuss N.H. et al. (2022) [42]	US	Case report	2022	Gastric GIST	1	Partial gastrectomy	Intuitive Surgical Da Vinci SP®	3	NS	NS	NS	OT: 82 min; no IO complications, no PO complications. hospital discharge: POD 2
Sugita H. et al. (2020) [43]	Japan	Case report	2020	Gastric GIST	1	Partial gastrectomy	Asensus Senhance®	2 (+1 AP)	NS	NS	NS	OT: 117 min, DT: 11 min; CT: 59 min; no BL; no complications; hospital discharge: POD 8
Cui H. et al. (2022) [44]	China	Case report	2022	Antral adenoca	1	total gastrectomy with D2 lymphadenectomy	Intuitive Surgical Da Vinci SP®	3 (+1 AP)	2	NS	NS	OT: 245 min, DT: 10 min; BL: 225 ml; no complications; hospital discharge: POD 8
Cubisino A. et al. (2022) [45]	US	Case series	2022	GERD	2	1 Nissen fundoplication; 1 redo Nissen flundoplication	Intuitive Surgical Da Vinci SP®	3	1	275 SP procedure, extensive experience in single site and robotic	NS	OT: 147 min, DT: 2.5 min; CT: 144.5 min; BL 10 ml; no IO complications; hospital discharge POD 2.5
Non-comparative studies												
Puntambekar S. et al. (2022) [85]	India	Retrospective analysis of a prospectively collected database	22 month (Aug 2019-Jun 2021)	Oesophagel neoplasia	57	transthoracic oesophagectomy	CMR Versius®	2 (+1 AP)	1	Oncologic surgeon with extensive robotic experience (>800 cases)	Team member: 3.5 day training program	OT: 230 min (47-330); BL: <500cc; Clavien-Dindo≥3 : 0; no reoperations; hospital discharge: POD 6
Schmitz R. et al. (2020) [86]	Germany	Retrospective analysis of a prospectively collected database	26 month (Mar 2017-Jul 2019)	GERD	18	Nissen fundoplication	Asensus Senhance®	2 (+1 AP + 1 liver retractor)	4	>400 procedures	Cadaver lab and supervision + training sessions. Inguinal hernia before the first Nissen	OT: 95,5 min (68-194); DT: 7 min (4-14); CT: 74 min (52-172); conversion to laparoscopy: 5.5%; Clavien-Dindo≥3: 5.5%

Li W. et al (2021) [84]	China	Case series	13 mont (Mar 2018-Apr 2019)	Morbid obesity	7	Sleeve gastrectomy	Wego MicroHand S II	2 (+ 1 AP)	NS	Sufficient training (NS)	NS	OT: 160 min; BL: 21cc; no Clavien-Dindo≥3; hospital discharge: POD 4; weigh loss at 3 mos: 63%

All the reported values are absolute or median if not specified. NS: not specified; IO: intraoperative; PO: postoperative; OT: operative time; CT: console time; DT: docking time; AP: assistant port; BL: blood loss; LOS: length of stay; Adenoca: adenocarcinoma; GIST: gastrointestinal stromal tumor; GERD: gastroesophageal reflux disease.
