# Supplementary material for: New Robotic Platforms in General Surgery: What’s the Current Clinical Scenario?
Source: Medicina (Kaunas). 2023 Jul 7;59(7):1264. doi: 10.3390/medicina59071264 (PMC10386395; doi:10.3390/medicina59071264)
Supplement: Supplementary file 1 [file medicina-59-01264-s001.zip › Table S7.pdf]

Table S7. Breast surgery

References	Country	Study design	Time period	Surgical indication	No. of pts/ procedures	Type of intervention	Robotic platform	No. of operative [illegible]	Surgeons involved	Surgeon previous experience	Surgical Team training	Main results
Case report/Technical notes												
Park H.S. et al. (2020) [40]	South Korea	Case report	2019	breast cancer	1	bilateral nipple-sparing mastectomy with one-stage reconstruction	Intuitive Surgical Da Vinci SP®	3	2	NS	Surgeons practiced on 6 breasts of 3 cadavers	OT: 170 min (left) and 180 min (right); DT: 5 min; CT: 93 min (left) and 109 min (right); total OT including reconstruction: 619 min; no Clavien-Dindo≥3; hospital discharge: POD 15; drain ablation: POD 26 (left) and POD 24 (right)
Joo O.Y. et al. (2020) [41]	South Korea	Case report	2020	breast cancer	2	1 nipple-sparing mastectomy with one-stage reconstruction (and acellular dermal matrix adoption); 1 nipple-sparing mastectomy with tissue expander	Intuitive Surgical Da Vinci SP®	3	1	NS	NS	OT (one-stage) : 6 hours; OT (two-stage): 2 hours; no Clavien-Dindo≥3
Non-comparative studies												
Go J. et al. (2022) [80]	South Korea	Retrospective analysis	36 mos (2018-2021)	breast cancer	70 patients (81 procedures)	53 nipple-sparing mastectomy with one-stage reconstruction; 16 nipple-sparing mastectomy with DIEP flap; 11 nipple-sparing mastectomy with tissue expander; 1 nipple-sparing mastectomy with LD flap	Intuitive Surgical Da Vinci SP®	3	NS	NS	NS	OT: 259 min (unilateral), 407 min (bilateral); CT: 48 min (unilateral), 110 min (bilateral); incision length: 45 mm; Clavien-Dindo≥3: 7.4%; drain ablation: 13 days

All the reported values are absolute or median if not specified. NS: not specified; IO: intraoperative; PO: postoperative; OT: operative time; CT: console time; DT: docking time; LOS: length of stay.
